# Laminin γ1 chain peptide, C-16 (KAFDITYVRLKF), promotes migration, MMP-9 secretion, and pulmonary metastasis of B16–F10 mouse melanoma cells

**DOI:** 10.1038/sj.bjc.6600187

**Published:** 2002-04-08

**Authors:** Y Kuratomi, M Nomizu, K Tanaka, M L Ponce, S Komiyama, H K Kleinman, Y Yamada

**Affiliations:** Craniofacial Developmental Biology and Regeneration Branch, National Institute of Dental and Craniofacial Research, NIH, Bethesda, Maryland, MD 20892, USA; Department of Otorhinolaryngology, Faculty of Medicine, Kyushu University, 3-1-1 Maidashi, Higashi-ku, Fukuoka 812-8582, Japan; Graduate School of Environmental Earth Science, Hokkaido University, Sapporo 060-0810, Japan; Department of Orthopedics, Faculty of Medicine, Kyushu University,3-1-1 Maidashi, Higashi-ku, Fukuoka 812-8582, Japan

**Keywords:** laminin, peptide, metastasis, migration, adhesion

## Abstract

Laminin-1, a heterotrimer of α1, β1, and γ1 chains specific to basement membrane, promotes cell adhesion and migration, proteinase secretion and metastases of tumour cells. Several active sites on the α1 chain have been found to promote B16–F10 melanoma lung colonisation and here we have determined whether additional tumour promoting sites exist on the β1 and γ1 chains. Recently, we have identified novel cell adhesive peptides derived from laminin β1 and γ1 chains by systematic screening of synthetic peptides. Nine β1 peptides and seven γ1 peptides active for cell adhesion were tested for their effects on experimental pulmonary metastases of B16–F10 mouse melanoma cells *in vivo*. The most active adhesive peptide derived from the γ1 chain globular domain, C-16 (KAFDITYVRLKF), significantly enhanced pulmonary metastases of B16–F10 cells, whereas no other peptides showed enhancement. C-16 also stimulated migration of B16–F10 cells in the Boyden chamber assay *in vitro*. Furthermore, C-16 significantly induced the production of MMP-9 from B16–F10 cells. These results suggest that this specific laminin γ1 chain peptide has a metastasis-promoting activity and might be a new molecular target of anti-cancer treatment.

*British Journal of Cancer* (2002) **86**, 1169–1173. DOI: 10.1038/sj/bjc/6600187 www.bjcancer.com

© 2002 Cancer Research UK

## 

Laminin-1 is part of a family of glycoproteins specific to basement membrane. It has multiple biological activities including promoting cell adhesion, migration, differentiation, neurite outgrowth and tumour cell malignancy (Timpl *et al*, 1979; Kleinman *et al*, 1985; Martin and Timpl, 1987; Timpl, 1989). Laminin-1 enhances the metastatic phenotype of tumour cells (Terranova *et al*, 1982, 1984; Barsky *et al*, 1984). In addition, laminin-1 induces production of collagenase IV (Turpeenniemi-Hujanen *et al*, 1986), urokinase-type plasminogen activator, and the 92-kDa matrix metalloproteinase (MMP-9) (Khan and Falcone, 1997) *in vitro*.

Several biologically active sites on mouse laminin-1 that affect tumour cells have been previously identified using synthetic peptides (Yamada and Kleinman, 1992). YIGSR on the β1 chain promotes tumour cell adhesion and migration (Graf *et al*, 1987a,b; Iwamoto *et al*, 1988) and inhibits experimental pulmonary metastases of mouse melanoma cells and angiogenesis (Iwamoto *et al*, 1987; Sakamoto *et al*, 1991). IKVAV on the α1 chain promotes cell adhesion, tumour growth, angiogenesis, collagenase IV activity by tumour cells, and experimental metastases as well as plasminogen activator activation (Grant *et al*, 1989; Tashiro *et al*, 1989; Kanemoto *et al*, 1990; Stack *et al*, 1991; Nomizu *et al*, 1992).

Recently, we have systematically screened a large set of overlapping synthetic peptides covering the whole mouse laminin-1 for their cell adhesive activities. Five peptides from the G-domain of α1 chain (Nomizu *et al*, 1995), 12 peptides from the γ1 chain (Nomizu *et al*, 1997), 21 peptides from the short and long arms of α1 chain (Nomizu *et al*, 1998), and 12 peptides from the β1 chain (Nomizu *et al*, 2000) were found to have significant cell adhesive activity. In addition, several peptides from the laminin-1 have been identified to be active for angiogenesis (Malinda *et al*, 1999; Ponce *et al*, 1999), acinar formation of salivary gland (Hoffman *et al*, 1998), and neurite outgrowth (Powell *et al*, 2000).

We previously identified several laminin α1 peptides that influence the metastatic activities of B16–F10 melanoma cells. AG-73 peptide from the α1 G-domain causes liver metastases (Kim *et al*, 1998), A-13 peptide from the N-terminal globule promotes an increase in pulmonary metastases (Kuratomi *et al*, 1999), and the AG-73 peptide (LQVQLSIR) promotes increased lung colonies and liver metastases. Here we report effects of adhesive peptides from the β1 and γ1 chains on experimental pulmonary metastases of B16–F10 cells *in vivo*. We have screened other adhesive peptides from β1 and γ1 chains for metastatic activity. We found that one of the peptides derived from the short arm of γ1 chain, C-16 (KAFDITYVRLKF), significantly enhanced pulmonary metastases of B16–F10 cells. This peptide also stimulated the migration of B16–F10 cells in the Boyden chamber assay. In addition, C-16 significantly induced production of MMP-9 by B16–F10 cells.

## MATERIALS AND METHODS

### Preparation of synthetic peptides

Cell adhesive peptides from the laminin β1 and γ1 chain and control peptides were synthesised and purified by high performance liquid chromatography as previously described (Nomizu *et al*, 1997). The purity and identity of the peptides were confirmed by an analytical HPLC and a Sciex API IIIE triple quadruple ion spray mass spectrometer.

### Cells and culture

B16–F10 mouse melanoma cells (Fidler and Nicolson, 1976) (a gift of Dr IJ Fidler, Houston, TX, USA) were maintained in Dulbecco's modified Eagle's medium (DMEM; Life Technologies, Inc.) containing 10% foetal bovine serum (FBS; Hyclone, UT, USA), 100 units ml^−1^ penicillin, 100 μg ml^−1^ streptomycin (Life Technologies, Inc.).

### *In vivo* experimental pulmonary metastasis assay

For the *in vivo* experimental pulmonary metastasis assay, B16–F10 cells were detached by 0.02% EDTA in phosphate buffered saline (Versene, Life Technologies, Inc.) and suspended in DMEM (1 000 000 cells ml^−1^). The cell suspension (0.2 ml) was injected into the tail veins of female C57BL6/N mice (9–12 weeks old). 0.2 mg of cell adhesion peptides (1 mg ml^−1^ in DMEM) was also intravenously injected within 10 min after the cell injection to exclude cell aggregations by mixture of peptide and cells. The mice were sacrificed 16 days after injection. The lungs were removed, and the number of visible colonies on the surface of the lungs was counted. When many colonies were formed and the number of the colonies could not be counted correctly because of the fusion of the colonies, the number was scored as 500. Five mice were used for each peptide. Duplicate experiments gave similar results.

All animals were maintained in filter top micro isolator cages and provided with sterile water and food under conditions complying with National Institute of Health (NIH) regulations. All manipulations of experimental animals were conducted in a laminar flow hood using strictly controlled procedures adhering to the UKCCCR Guidelines for the Welfare of Animals in Experimental Neoplasia to minimise stress or suffering (UKCCCR, 1998).

### *In vitro* migration assay

Migration of B16–F10 cells through polycarbonate filters was assayed using 48 well chemotaxis chambers (modified Boyden chamber, Neuro Probe, MD, USA) as described previously (Kuratomi *et al*, 1999). The lower wells of the chamber were loaded with DMEM containing 0.1% bovine serum albumin (BSA, Sigma) (DMEM/BSA) and 100 μg ml^−1^ of peptide. Versene-detached B16–F10 cells (50 000 cells per 50 μl in DMEM/BSA) were placed in the upper wells. After a 5 h incubation, cells on the lower surface of the filter were stained with Diff-Quik (Baxter, IL, USA), and counted under a microscope. Each peptide was tested in triplicate and each experiment was repeated at least twice.

### Zymography

B16–F10 cells (2 500 000 cells) were plated onto 150 mm dishes with complete media. After 24 h, the media were replaced with serum-free DMEM containing various concentrations of peptides. The conditioned media were collected after a 16 h incubation at 37°C in 5% CO_2_ and concentrated 50 times by using Centriprep 10 (Amicon). Equal aliquots of the conditioned media (20 μl per lane) were electrophoresed on 10% polyacrylamide gels containing 0.2% gelatin. The gels were washed with 10 mM Tris-HCl (pH 7.4) containing 2.5% Triton-X for 30 min, followed by two changes of 10 mM Tris-HCl (pH 7.4) for 30 min. After an overnight incubation in 50 mM Tris-HCl (pH 8.0) containing 5 mM CaCl_2_ and 1 mM ZnCl_2_ at room temperature, Coomassie blue was added to visualise the digested gelatin bands.

## RESULTS

### Effect of cell adhesive laminin β1 and γ1 chain peptides on experimental pulmonary metastasis of B16–F10 mouse melanoma cells

Nine peptides active for cell adhesion from the mouse laminin β1 chain (Nomizu *et al*, 2000) and seven peptides from the γ1 chain (Nomizu *et al*, 1997) were tested for their effects on *in vivo* experimental pulmonary metastases of B16–F10 mouse melanoma cells (Table 1

**Table 1:**
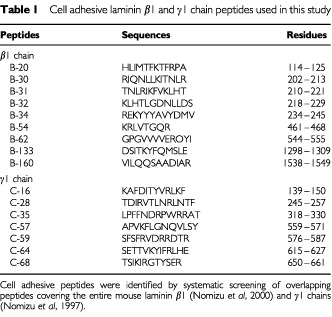
Cell adhesive laminin β1 and γ1 chain peptides used in this study

). In control experiments (without peptides), the average of 163 metastatic colonies was formed on the surface of the lung within 16 days after B16–F10 cells were injected into the mouse tail vein (Table 2

**Table 2:**
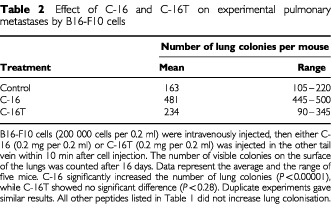
Effect of C-16 and C-16T on experimental pulmonary metastases by B16-F10 cells

). Peptide C-16 (KAFDITYVRLKF) from the N-terminal globular domain of the γ1 chain significantly enhanced the number of B16–F10 lung colonies (average of 482 colonies, Table 2 and Figure 1

**Figure 1:**
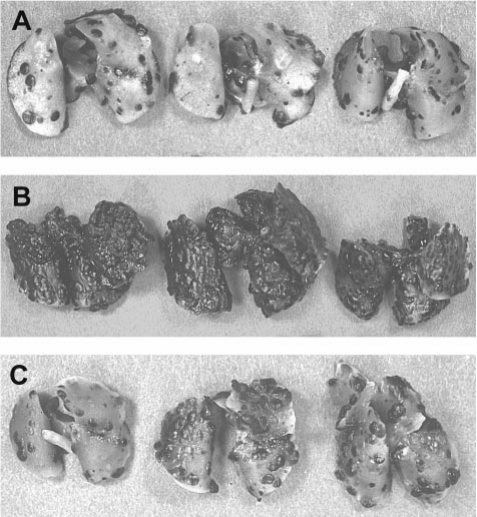
Appearance of the lung colonies formed by B16–F10 cells. Two hundred thousand cells per 0.2 ml were injected through the tail vein, then 0.2 mg per 0.2 ml of C-16 or the scrambled peptide of C-16, C-16T, was injected in the other tail vein within 10 min after the cell injection. Lungs were resected 16 days after the cell injection, then fixed in formalin, and photographed. C-16 (**B**) significantly promoted the formation of pulmonary metastases whereas C-16T (**C**) showed no enhancement as compared to control (**A**). Duplicate experiments gave similar results.

). No other peptides showed stimulation or inhibition of pulmonary metastases of B16–F10 cells (data not shown). A scrambled peptide of C-16, C-16T (FYAFKKITLVRD), slightly increased the number of B16–F10 lung colonies (average of 234 colonies with range from 90 to 345), but the difference was not significant (Table 2, Figure 1). These results suggested a sequence-specific enhancement of *in vivo* pulmonary metastases of B16–F10 cells by C-16.

### *In vitro* cell migration

**Figure 2:**
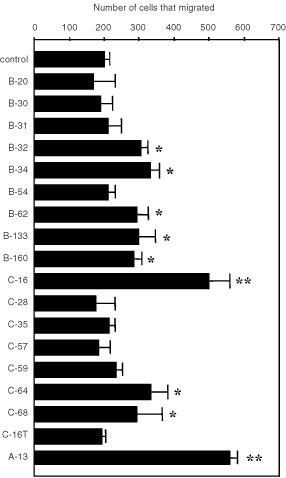
Effect of cell adhesive laminin β1 and γ1 chain peptides on B16–F10 cell migration. Migration of B16–F10 cells through the filters was assayed by using 48 well Boyden chambers. The upper chamber contained 50 000 cells and the lower chamber contained 100 μg ml^−1^ of peptide. After a 5 h incubation, the number of cells that migrated in the centre of each well was counted under the microscope. Data represent mean±s.d. of triplicate wells. Duplicate experiments gave similar results. *P<0.05; **P<0.01.

In order to study the mechanism of the enhancement of B16–F10 lung colonisation by C-16, we examined the activities of these 16 cell adhesive peptides for cell migration. Among the 16 cell adhesive peptides, 8 peptides (B-32, 34, 62, 133, 160; C-16, 64, 68) stimulated migration of B16–F10 cells, with C-16 having the strongest enhancement activity (Figure 2

). The migration–stimulatory activity of C-16 seemed to be comparable to that of peptide A-13 from the α1 chain which previously was shown to be highly active (Nomizu *et al*, 1998; Kuratomi *et al*, 1999). A control scrambled peptide, C-16T, showed no stimulatory activity of B16–F10 cell migration.

### Gelatin zymography

**Figure 3:**
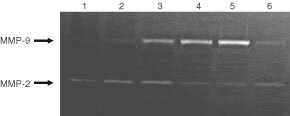
Effect of C-16 on activation of gelatinases. The conditioned media of B16–F10 cells incubated without peptide (lane 1) or with 2 μg ml^−1^ (lane 2), 5 μg ml^−1^ (lane 3), 10 μg ml^−1^ (lane 4), 20 μg ml^−1^ (lane 5) of C-16, or 20 μg ml^−1^ of C-16T (lane 6) were harvested and electrophoresed on 10% SDS gel containing 1% gelatin. C-16 stimulated the activity of MMP-9 (92-kDa gelatinase) in a dose-dependent manner, whereas C-16T showed no stimulation.

Because matrix metalloproteinases (MMPs) have been implicated in the metastatic process of tumour cells, we measured MMP production from B16–F10 cells in the gelatin zymography assays (Figure 3

). C-16 enhanced the production of MMP-9 (92 kDa gelatinase) in a dose-dependent manner. With 20 μg ml^−1^ of C-16, MMP-9 production was increased by approximately eight-fold over that observed with the untreated control. MMP-2 production was not stimulated by C-16. No other cell adhesive peptides from the β1 and γ1 chains stimulated the production of MMPs (data not shown).

## DISCUSSION

Laminin-1 has been shown to promote the metastatic activity of melanoma cells (Terranova *et al*, 1982, 1984). We previously reported that several peptide sequences including RQVFQVAYIIIKA (A-13), IKVAV, and LQVQLSIR (AG-73) from the laminin α1 chain have such activity (Kanemoto *et al*, 1990; Kim *et al*, 1998; Kuratomi *et al*, 1999). In this report, we identified a new site, C-16 (KAFDITYVRLKF), on the laminin γ1 chain which promotes lung colonisation of B16–F10 cells in an experimental pulmonary metastasis model in mice.

C-16 enhanced B16–F10 colonization about three-fold compared with control. C-16 appears to be the only site on the γ1 chain of laminin-1 active for lung colonisation and no site on the β1 chain was found to have this activity. C-16-mediated metastasis promoting activity is comparable to that of A-13 and IKVAV. Since laminin-1 is a highly potent promoter of the malignant phenotype, it is not surprising that multiple sites for metastases have been identified.

The mechanism by which A-13 and C-16 increase lung colonisation is not known. Cells must adhere, migrate, invade (protease activity), proliferate, and generate blood supply in order to form a growing metastatic lesion. A-13 and C-16 have been found to promote all of these activities except cell proliferation. The role of A-13 and C-16 in angiogenesis in the metastatic lesion has not been tested. We did not examine the vascularity of the lung lesions and it is unlikely that A-13 and C-16 persists in the circulation or tissue for very long after injection. These are small molecules which are cleared very rapidly from the circulation. We speculate that A-13 and C-16 may enhance lung metastases by increasing the invasion activity of the cells.

Laminin-1 stimulates cell migration (Kleinman *et al*, 1985) and laminin α1 peptides with metastasis-promoting activity also have activity for the migration of B16–F10 cells (Kuratomi *et al*, 1999). We have shown here that five β1 peptides and three γ1 peptides stimulated migration of B16–F10 cells, with C-16 being most active and comparable to A-13. Thus, laminin-1 has multiple active sites for cell migration, which may function cooperatively.

Matrix metalloproteinases have been thought to be critical in tumour invasion and metastases as well as in angiogenesis (Liotta, 1986; Liotta *et al*, 1991; Kohn and Liotta, 1995). C-16 showed strong stimulation of MMP-9 production by B16–F10 cells. The IKVAV peptide from the laminin α1 chain also induces MMP-1 and MMP-9 production from human monocyte/macrophage (Corcoran *et al*, 1995). AG-73 was also reported to stimulate MMP-9 activity of B16–F10 cells at the concentration of 50 μg ml^−1^ (Kim *et al*, 1998). C-16 is more potent in inducing MMP-9 production than AG-73. C-16 stimulated MMP-9 production nearly eight-fold at a concentration of 2 μg ml^−1^. This high activity of C-16 for MMP-9 production as well as for cell migration may explain its potent metastasis-promoting activity.

Recently, expression of the γ2 chain of laminin-5, an epithelial cell–specific laminin, was predominantly detected at the invasive front of cancer cells of the colon, pancreas, stomach and oesophagus (Pyke *et al*, 1995; Soini *et al*, 1996; Sordat *et al*, 1998; Koshikawa *et al*, 1999; Yamamoto *et al*, 2001). The laminin γ2 chain has been suggested to play a key role in the progression of human carcinomas. C-16 is located in the N-terminal globular domain of the laminin γ1 chain, and this domain is deleted in the γ2 chain. However, it is possible that the deleted N-terminal globular domain is co-expressed with laminin γ2 chain by the proteolytic cleavage of the γ2 chain and that the cleaved domain induces migration and metastases of tumour cells in human cancers. Indeed, Giannelli *et al* (1997) have reported that laminin-5 becomes active for promoting cell migration when cleaved by MMP-2. It is possible that these active fragments of laminin-1 promote cell migration when it is degraded similar to laminin-5.

**Figure 4:**
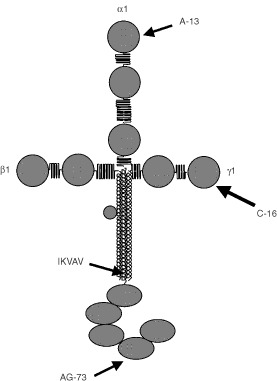
Novel metastasis-promoting peptides from the mouse laminin 1. AG-73 (Kim *et al*, 1998) and A-13 (Kuratomi *et al*, 1999) were found to promote experimental pulmonary metastases of B16–F10 cells. Previously, IKVAV was found to promote lung colonisation (Kanemoto *et al*, 1990). All peptides show stimulation of B16–F10 cell adhesion and migration activities. C-16, AG-73, and IKVAV stimulate 92-kDa gelatinase (MMP-9) activity of B16–F10 cells.

Four novel active sites (IKVAV, AG-73, A-13 and C-16) on mouse laminin-1 have been identified for B16–F10 cell adhesion, migration, MMP secretion, and metastases in this report and in previous studies (Figure 4

) (Kanemoto *et al*, 1990; Kim *et al*, 1998; Kuratomi *et al*, 1999). Interestingly, A-13 and C-16 are in homologous locations on their respective chains at the amino termini, binding to the same receptors and have similar activity (Ponce *et al*, 2001; and unpublished data). Of the 12 amino acids, three amino acids are identical and five are conserved. Preservation of sequences and activity suggest important functional sites. Degradation of laminin chains may also be important in exposure of these active sites. These active sites might be new molecular targets of anti-cancer treatments which prevent distant metastases of malignant tumours. Furthermore, specific antibodies to these sites and inhibitory peptides might lead to development of therapeutic agents for human malignant tumours.
